# Phage therapy: Awareness and demand among clinicians in the United Kingdom

**DOI:** 10.1371/journal.pone.0294190

**Published:** 2023-11-13

**Authors:** Emily A. Simpson, Helen J. Stacey, Ross J. Langley, Joshua D. Jones

**Affiliations:** 1 Medical Microbiology, Ninewells Hospital, NHS Tayside, Dundee, United Kingdom; 2 Public Health, Kings Cross Hospital, NHS Tayside, Dundee, United Kingdom; 3 Department of Paediatric Respiratory and Sleep Medicine, Royal Hospital for Children, Glasgow, United Kingdom; 4 School of Medicine, Dentistry & Nursing, University of Glasgow, Glasgow, United Kingdom; 5 Infection Medicine, Edinburgh Medical School: Biomedical Sciences, University of Edinburgh, Edinburgh, United Kingdom; Cornell University, UNITED STATES

## Abstract

Bacterial resistance or tolerance to antibiotics is costly to patients and healthcare providers. With the impact of antibiotic resistance forecast to grow, alternative antimicrobial approaches are needed to help treat patients with antibiotic refractory infections and reduce reliance upon existing antibiotics. There is renewed interest in bacteriophage (phage) therapy as a promising antimicrobial strategy. We therefore performed the first multi-specialty survey about phage therapy and the first such survey among clinicians in the United Kingdom. An anonymous 10-question survey of clinicians from medical and surgical specialties in two Scottish Health Boards was performed. The 90 respondents spanned 26 specialties and were predominantly consultants (73.3%). The respondents were concerned about antibiotic resistance in their clinical practice; 83 respondents estimated having seen 711 patients in the last 12 months whose infections were refractory to antibiotics (delaying or preventing resolution). Over half (58.8%) of the respondents had previously heard of phage therapy. Staphylococci, *Pseudomonas* and *E*. *coli* were identified as the highest cross-specialty priorities for the development of phage therapy. Together, 77 respondents estimated seeing 300 patients in the last 12 months for whom phage therapy may have been appropriate (an average of 3.9 patients per clinician). Most respondents (71.1%, n = 90) were already willing to consider using phage therapy in appropriate cases. Additional comments from the respondents affirmed the potential utility of phage therapy and highlighted a need for more information. The results of this survey demonstrate substantial demand for and willingness to use phage therapy in appropriate cases, both from individual clinicians and across specialties. Demand from a wide range of specialties illustrates the broad clinical utility of phage therapy and potential scope of impact. Widening access to phage therapy could deliver substantial clinical and financial benefits for patients and health authorities alike.

## Introduction

Antibiotic resistance is a major global health concern and was associated with an estimated 4.95 million deaths worldwide in 2019 [1]. The leading pathogens associated with resistance were *Escherichia coli*, *Staphylococcus aureus*, *Klebsiella pneumoniae*, *Streptococcus pneumoniae*, *Acinetobacter baumannii*, and *Pseudomonas aeruginosa*, with resistance among these organisms associated with 3.57 million deaths. The impact of antibiotic resistance is set to grow; it has been estimated that by 2050 antibiotic resistance could cause 10 million deaths each year, costing the world economy around US$100 trillion [2]. While antibiotic resistance refers to the carriage of resistance genes by bacteria, antibiotic tolerance afforded by biofilm production or phenotypic changes in bacterial cells also poses a significant health burden [3]. Antibiotic tolerance is thought to underpin many chronic infections which are sensitive to antibiotics but clinically refractory to antibiotic therapy [4]. Therefore, resistance and/or tolerance can underlie antibiotic refractory infections. Consequently, there is an urgent need for alternative antimicrobial strategies which will enable the treatment of antibiotic refractory infections and help reduce reliance on, and therefore conserve, existing antibiotics.

Bacteriophage (phage) therapy is a promising antimicrobial strategy. Phages are naturally occurring viruses that have evolved to infect and kill bacteria in a generally species-specific manner. Found wherever bacteria are, we exist in constant exposure to phages; for example, phages form a significant part of our commensal microbiota [5]. Phages were first used to treat bacterial infection in 1919, but a limited understanding of phages at the time and the mass production of antibiotics contributed to the disappearance of phage therapy from Western medicine [6]. Today, the antibiotic resistance crisis has driven renewed interest in phage therapy. Clinical and safety trials of phage therapy, administered by a variety of routes, have consistently demonstrated safety [7–20]. Similarly, a recent systematic review of observational clinical from data 2241 patients treated with phages since the year 2000 found that phage therapy was well tolerated with any adverse events mild [21]. While the efficacy of phage therapy has not been consistently demonstrated by clinical trials, this is considered to reflect methodological rather than mechanistic shortcomings [22]. In contrast, there is a substantial and compelling body of observational data supporting the efficacy of phage therapy among patients with difficult-to-treat infections [21]. In recent years the American Antibiotic Resistance Leadership Group and Health Improvement Scotland have recommended that phage therapy be considered for difficult-to-treat bacterial infections [23, 24].

Currently, there are no licensed phage therapy products in the UK and phage therapy may only be considered for use as an unlicensed medicine when licensed alternatives (e.g. antibiotics) are not meeting clinical needs [25]. Patients for whom unlicensed phage therapy may be appropriate include those with: antibiotic resistant infections; antibiotic susceptible but clinically recalcitrant chronic infections; reasonably foreseen acute risk to life or limb despite appropriate antibiotic treatment; other patient-specific factors that preclude the use of appropriate antibiotics (e.g. renal failure, allergy, drug-drug interactions or intolerable side effects) or cases where further medical intervention is preferred to surgery (e.g. in high-risk surgical candidates) [24]. This represents a disparate group of unique patients that is challenging to quantify through prevalence data.

Phage therapy has the potential to transform the way we treat bacterial infections. While reduced morbidity and mortality will benefit patients, the potential savings offered to health authorities by successful implementation of phage therapy are substantial. Unsurprisingly, there is growing interest in phage therapy among clinicians. There have been two previous surveys of clinicians about phage therapy, undertaken in Canada and Australia, but no similar surveys have been undertaken in our context [26, 27]. Given the recent Health Improvement Scotland recommendation, it is important to capture and demonstrate concern about antibiotic resistance and the magnitude of interest in phage therapy to inform policy decisions. It is also important to address the question of how many patients the use of unlicensed phage therapy could potentially benefit. We therefore undertook a survey of clinicians across two Scottish Health Boards to investigate the awareness of and demand for phage therapy.

## Methods

### Survey design and distribution

An anonymous 10-question survey was designed (S1 File). The survey was for doctors of any grade in National Health Service (NHS) Tayside and NHS Greater Glasgow and Clyde (NHS GG&C). The survey questions were prefaced by brief background information about phage therapy and the survey itself. A mixture of tick-box, rating scale and free-text responses were used. Questions one and two asked respondents their grade (e.g. consultant or specialty trainee) and specialty. Questions three and four addressed antibiotic resistance, asking respondents to rank their concern about antibiotic resistance on a scale of one to five and then to estimate the number of patients they had treated in the last year whose infections were refractory to antibiotics (delaying or preventing resolution). Questions five to nine addressed phage therapy. Respondents were first asked whether they had heard of phage therapy prior to this survey. Next, respondents were asked to rank nine bacterial genera/species by priority for the development of phage therapy. The nine genera/species included the six leading pathogens associated with resistance identified by Murray and colleagues. Three additional, often antibiotic resistant, genera were included (*Burkholderia*, *Enterococcus* and mycobacteria). Respondents were then given a free-text opportunity to state any other pathogens for which they considered phage therapy should be developed. Recognising that many respondents may be unfamiliar with phage therapy, brief information about the unlicensed nature of phage therapy in the UK and potentially appropriate clinical scenarios was then presented. Respondents were then asked to estimate the number of patients treated in the last year for whom phage therapy may have been appropriate (i.e. for whom antibiotics were not meeting their clinical needs). Next, respondents were asked whether they would consider phage therapy, if appropriate, for their patients. Finally, a free-text box was provided for additional comments. The survey was kept brief to increase the likelihood of responses from time-poor respondents. The survey was designed to take around four minutes to complete.

The survey was presented using Microsoft Forms and was distributed in two Scottish Health Boards: NHS Tayside (08/03/23 to 26/05/23) and NHS GG&C (17/04/23 to 26/05/23). Respondents were invited by e-mail and in NHS GG&C the survey was advertised on an internal staff newsletter. Respondents were encouraged to forward the survey onto colleagues, consequently precluding calculation of a response rate. Informed consent was implied by completion of the survey. This service development survey was undertaken as part of a biomedical scientist’s training portfolio. Internal NHS approvals were obtained in NHS Tayside from the relevant training manager and Associate Medical Director and in NHS GG&C from the Patient Experience and Public Involvement Team.

### Data interpretation

The survey data were downloaded from Microsoft Forms into Microsoft Excel, where the data were curated and charts and descriptive statistics produced. Any responses identified as not being from a doctor of any grade were excluded. As free-text questions, the same information about grade or specialty could be provided by respondents in different forms. Grades were therefore curated into three categories: consultant, registrar or other. For specialties, synonymous responses (e.g. internal medicine, general medicine and acute internal medicine) were grouped together. Two additional groupings were: intensive care medicine, critical care and anaesthetics; dermatology and dermatology with general practice. Estimations of patient numbers were also free-text responses and consequently the data obtained required curation. Where a range was provided (e.g. 5–10) the middle value was taken, where this was a decimal (e.g. 7.5) the figure was rounded to the nearest patient. Where qualifiers (e.g. more than or less than or around) were provided with numerical answers (e.g. around 10), these were discounted and the numerical figure provided was taken. Non-numerical answers that were written values (e.g. none) were accepted and converted into numerical data. Non-numerical answers that were not written values (e.g. unable to estimate a number but many) were classified as uninterpretable and discounted. Synonymous responses (e.g. unsure, don’t know) were also collated for responses to the question about other organisms respondents would like phage therapy for. A thematic analysis of the additional comments was undertaken; the spelling and grammar of comments were minimally curated.

## Results

### Respondents

A total of 91 responses were received, 42 from NHS GG&C and 49 from NHS Tayside. The raw data from both Health Boards were collated and curated (S2 File). One response was identified as not being from a doctor of any grade and was excluded. Of the remaining 90 responses, 73.3% were consultants (n = 66), 14.4% were registrars (n = 13) and 12.2% were other grades of doctor (n = 11). Responses were obtained from 26 medical or surgical specialties (Table 1). Paediatric specialties, including neonatology, were considered together for subsequent analyses, which therefore represented 20 specialties (22.2%).

**Table 1:** Respondent specialties (n = 90).

Specialty	N	%
Paediatric medicine	13	14.4
Respiratory medicine	10	11.1
Diabetes & endocrinology	6	6.7
Obstetrics and gynaecology	6	6.7
Orthopaedics	6	6.7
Dermatology	5	5.6
General/acute medicine	4	4.4
Haematology	4	4.4
Medicine of the elderly	4	4.4
Neonatology	4	4.4
Infectious diseases	3	3.3
Paediatric respiratory medicine	3	3.3
Renal medicine	3	3.3
Anaesthetics / intensive care/ critical care	3	3.3
Ear, nose and throat	2	2.2
Microbiology	2	2.2
Plastic surgery	2	2.2
Vascular surgery	2	2.2
Orthodontics	1	1.1
General practice	1	1.1
Genitourinary medicine	1	1.1
Neurosurgery	1	1.1
Paediatric infectious diseases	1	1.1
Community paediatrics	1	1.1
Paediatric neurology	1	1.1
Paediatrics and neonatology	1	1.1
Total	90	100

### Antibiotic resistance

**Fig 1:**
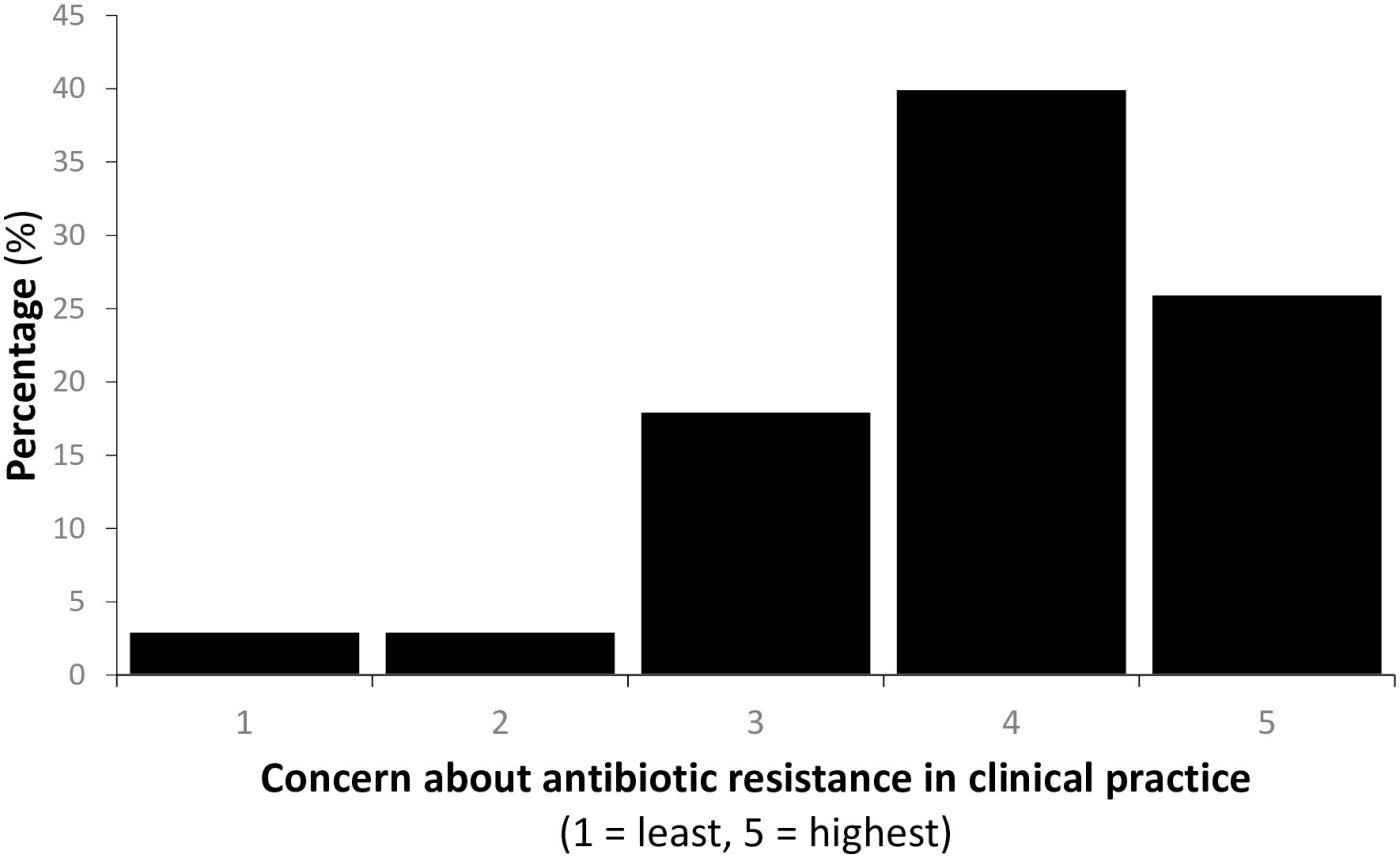
Concern about antibiotic resistance in clinical practice (n = 90).

Fig 1 shows that overall concern about antibiotic resistance in clinical practice was high (average = 3.9/5), with 73.3% (n = 66) scoring concern as four or five out of a possible five. Table 2 shows the specialties of the respondents who scored concern as a four or five. The respondents which rated antibiotic resistance as being of least concern included community paediatrics and orthodontics.

**Table 2:** The specialties of respondents who rated concern about antibiotic resistance as a 4 or 5 (n = 66).

Specialty	N
Paediatrics (all subspecialties)	13
Respiratory medicine	10
Diabetes & endocrinology	5
Obstetrics & gynaecology	5
Medicine of the elderly	4
Trauma & orthopaedics	4
Acute / general medicine	3
Anaesthetics / ICU / critical care	3
Haematology	3
Infectious diseases	3
Renal medicine	3
Microbiology	2
Neonatology	2
Vascular surgery	2
Dermatology	1
Ear, nose and throat	1
Genitourinary medicine	1
Plastic surgery	1
Total	66

The respondents were then asked to estimate the number of patients treated in the last year whose infections were refractory to antibiotics, delaying or preventing resolution of infection. Seven of the 90 responses were classed as uninterpretable. The remaining 83 clinicians collectively estimated that they had seen 711 patients with antibiotic refractory infections in the last 12 months, an average of 8.6 patients per clinician. The per specialty estimations (with all paediatric specialties considered together) are shown in Table 3. Among the specialties with five or more respondents, diabetes and endocrinology had the highest estimated rate of antibiotic refractory infections at 15.7 patients per clinician.

**Table 3:** Per specialty estimations of the number of patients with antibiotic refractory infections in the last 12 months.

Specialty	N	Total	Patients per clinician
Paediatrics (all subspecialties)	21	106	5.0
Respiratory medicine	9	127	14.1
Diabetes & endocrinology	6	94	15.7
Obstetrics & gynaecology	6	14	2.3
Trauma & orthopaedics	6	14	2.3
Dermatology	5	40	8.0
Acute / general medicine	4	51	12.8
Haematology	4	10	2.5
Anaesthetics / intensive care/ critical care	3	40	13.3
Infectious diseases	3	39	13.0
Renal medicine	3	10	3.3
Microbiology	2	80	40.0
Medicine of the elderly	2	53	26.5
Ear, nose and throat	2	10	5.0
Plastic surgery	2	6	3.0
General practice	1	10	10
Vascular surgery	1	6	6
Genitourinary medicine	1	1	1
Neurosurgery	1	0	0
Orthodontics	1	0	0
Total	83	711	-

### Awareness of and demand for phage therapy

There was a high level of awareness of phage therapy among the 90 respondents, with 58.8% (n = 53) having heard of phage therapy before taking the survey, while 36.6% (n = 33) had not previously heard of phage therapy and 4.4% (n = 4) were unsure.

**Fig 2:**
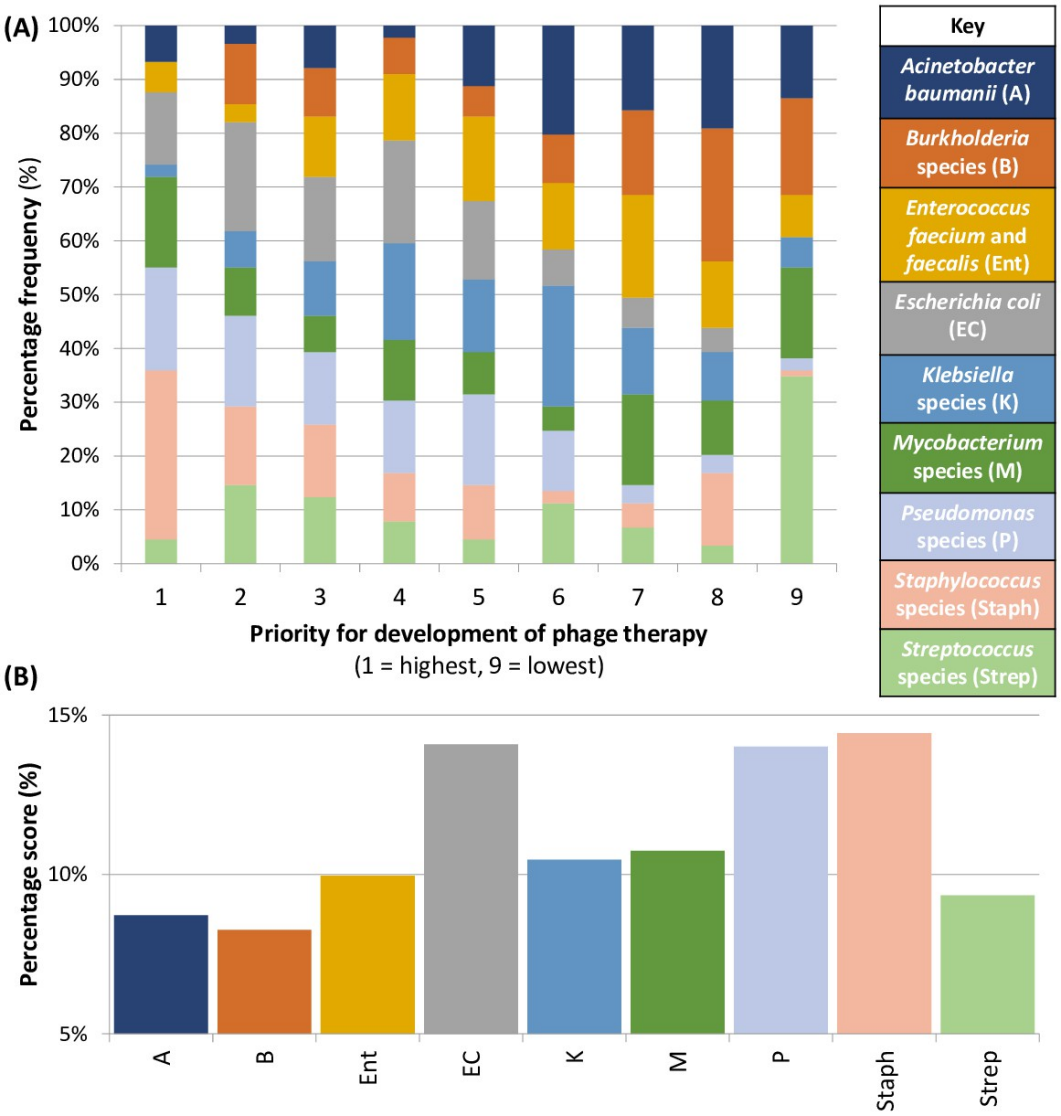
Respondent ranking of nine genera/species in order of priority for development of phage therapy (n = 89). (A) The percentage frequency of each genus/species at each of the nine ranking positions. (B) The percentage of the total score available across the ranking scale.

**Fig 3:**
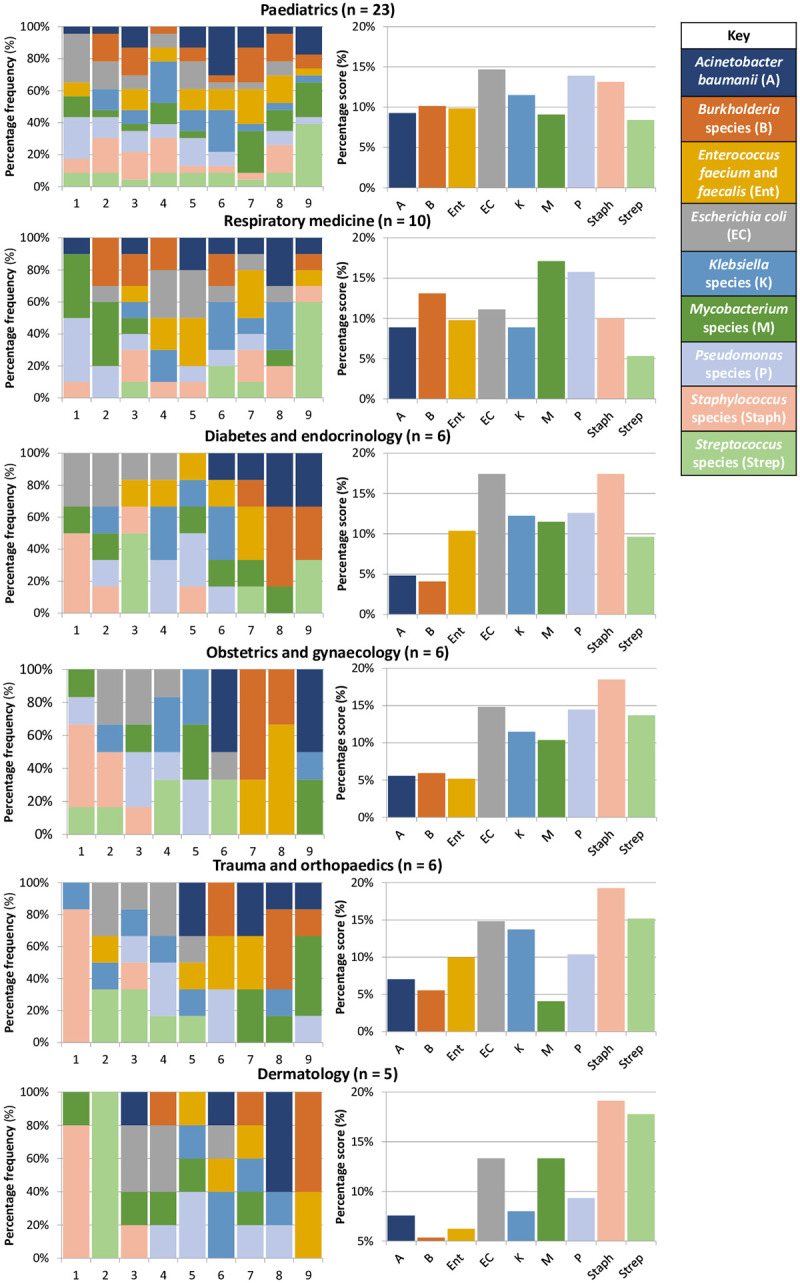
Respondent ranking of nine genera/species in order of priority for development of phage therapy among specialties with five or more respondents.

Next, clinicians were asked to rank nine genera/species of bacterial pathogen by priority for development of phage therapy. One respondent noted that they ‘had no idea’ and had ‘guessed’ the order; this response was excluded from further analysis. Fig 2A shows the percentage frequency with which each genus/species was ranked at each of the nine positions by the remaining 89 respondents. To investigate the significance of each genus/species across the whole ranking scale a scoring system was used whereby a maximum of nine points was awarded to each genus/species when it was ranked by a respondent as highest priority, through to one point when it was ranked least important. The percentage scored by a genus/species of the total points available across the scale therefore indicated its significance. The results in Fig 2B showed that staphylococci, *E*. *coli* and *Pseudomonas* were the three genera/species which scored as the highest priorities for phage therapy across all specialties. Next, we inspected the per specialty trends; data for specialties with five or more respondents are shown in Fig 3. Application of the scoring system to specialty-specific datasets revealed the significance of each genus/species in each specialty (Fig 3). This showed that for paediatrics (n = 23) *E*. *coli*, *Pseudomonas* and staphylococci were the highest priorities. Perhaps unsurprisingly, for respiratory medicine physicians (n = 10) *Mycobacterium*, *Pseudomonas* and *Burkholderia* were of greatest importance. In contrast, for diabetes and endocrinology (n = 6) and obstetrics and gynaecology (n = 6), *E*. *coli* and staphylococci scored most highly. Whereas for trauma and orthopaedics (n = 6) or dermatology (n = 5), staphylococci and streptococci were of highest priority.

Mindful that the ranking question offered a limited range of pathogens, respondents were asked if there were any other pathogens they would like phage therapy for. This was an optional question and most had either no response (n = 53, 58.9%), said that there were no additional pathogens (n = 19, 21.1%) or did not know (n = 7, 7.8%). The remaining responses are shown in Table 4, with *Clostridium* (n = 3) and *Stenotrophomonas* (n = 2) the only genera mentioned more than once.

**Table 4:** Responses when asked if there were other pathogens the respondents would like phage therapy to be developed for.

Response	N	%
No response	53	58.9
No	19	21.1
Don’t know	7	7.8
Clostridium	3	3.3
Stenotrophomonas	2	2.2
Bacillus	1	1.1
Resistant Gram negative organisms	1	1.1
Fungi	1	1.1
Group B Streptococcus	1	1.1
Neisseria gonorrhoeae	1	1.1
Staphylococcus aureus	1	1.1
Total	90	100

Unlicensed phage therapy is only available when licensed medicines are not fulfilling clinical needs. Even within a single specialty the unique nature of such cases makes it challenging, if not impossible, to obtain precise prevalence data about the potential numbers of patients for whom unlicensed phage therapy may be suitable. After outlining the circumstances in which unlicensed phage therapy may be appropriate, we therefore asked respondents to estimate the number of patients they had treated in the last year who may have been eligible for phage therapy. Among the 90 responses, 13 were classed as uninterpretable. Together, the remaining 77 clinicians estimated they had seen 300 patients in the last 12 months for whom phage therapy may have been appropriate: giving an average of 3.9 patients per clinician. In NHS Tayside, 40 clinicians estimated a total of 116 potential phage patients from the last 12 months, while in NHS GG&C the estimated total was 184 potential patients from 37 clinicians. Per specialty estimations are shown in Table 5.

**Table 5:** Per specialty estimations of the number of patients that may have been suitable for phage therapy in the last 12 months.

Specialty	N	Total	Patients per clinician
Diabetes & endocrinology	5	47	9.4
Respiratory medicine	9	47	5.2
Paediatrics (all subspecialties)	21	46	2.2
Acute / general medicine	4	30	7.5
Dermatology	4	28	7.0
Anaesthetics / intensive care/ critical care	2	24	12.0
Microbiology	2	20	10.0
Infectious diseases	3	12	4.0
Trauma & orthopaedics	5	12	2.4
Renal medicine	3	8	2.7
Haematology	4	6	1.5
Obstetrics & gynaecology	4	6	1.5
Medicine of the elderly	2	5	2.5
Ear, nose and throat	2	4	2.0
Vascular surgery	1	3	3.0
Genitourinary medicine	1	1	1.0
Plastic surgery	2	1	0.5
General practice	1	0	0.0
Neurosurgery	1	0	0.0
Orthodontics	1	0	0.0
Total	77	300	-

The respondents were then asked whether they would consider using phage therapy in an appropriate case. Most respondents said they would consider using phage therapy (n = 64/90, 71.1%) and 27.8% (n = 25) were unsure. Only one respondent (1.1%, paediatrics) said they would not consider phage therapy.

Finally, the respondents were provided with an optional additional comments question. Of the 90 respondents, 27 provided additional comments (30.0%). Of these 27 responses, 17 were provided from respondents who had indicated in the previous question that they would consider using phage therapy, while 10 additional comments were from respondents who had answered unsure. The additional comments were subjected to a thematic analysis and the comments and themes identified are shown in Table 6. Five themes were identified among the comments. The potential utility of phage therapy for clinical practice was the most common theme, apparent in 13 of the 27 comments. A desire for more information about phage therapy was the next most frequent theme, expressed by 11 respondents. Seven of the 11 respondents who expressed an interest in further information answered unsure when asked whether they would consider phage therapy. Six respondents expressed concern about current or future levels of antibiotic resistance in their clinical practice. Six respondents showed prior awareness of phage therapy from anecdotal, media, talks or literature sources; all had answered ‘yes’ when asked about awareness of phage therapy prior to this survey. Four respondents remarked that they would like multidisciplinary input into a decision to use phage therapy; three of these respondents specifically identified local infection services (e.g. infectious diseases or microbiology). No themes were identified in one response (‘all sounds very interesting’).

**Table 6:** Thematic analysis of additional comments.

Specialty	Would use phage therapy?	Comment	Phage therapy: useful	More information	Concerned about ABR	Prior awareness of phage therapy	Multidisciplinary team input
Infectious diseases	Yes	I have heard of anecdotes where phages may have helped individual patients.				✓	
Respiratory medicine	Yes	In my practice, I think phage therapy could be useful for non-tuberculous mycobacterial infection and resistant pseudomonas infection, all with regards to respiratory infection.	✓		✓		
Haematology	Unsure	Attractive option for dealing with infections, would need info re: safety to give phage therapy in profoundly immunocompromised haematology/bone marrow transplant setting	✓	✓			
Diabetes and endocrinology	Yes	Often very unwell co-morbid patients where surgical intervention carries significant risk and therefore additional therapies to help manage their disease burden would be most welcome.	✓				
Microbiology	Yes	Phage therapy for mycobacterial infection or other chronic lung infections and orthopaedic/prosthetic related infection most likely to be useful	✓				
Paediatric respiratory medicine	Unsure	Keen to know more about this interesting developing area and how it could potentially help our patients in the future.		✓			
Paediatrics	Yes	Would need specialist advice on use of this, but would be happy to consider if felt indicated	✓	✓			✓
Paediatrics	Unsure	Need a much greater understanding of its use, and direction from specialist team i.e. ID [infectious diseases] if was to use.		✓			✓
Neonatology	Yes	Several patients who have been colonised with an organism that has some antibiotic resistance but usually have an antibiotic that organism is sensitive to. Don’t recall any patients who had an organism that we had no antibiotics to treat however can envisage this may become an issue in the future.	✓		✓		
Neonatology	Unsure	This is a therapy I am unfamiliar with and would need to review the evidence to feel comfortable with use, especially in the field of neonates		✓			
Diabetes and endocrinology	Unsure	I think I would need to learn more about phage therapy before being able to recommend/prescribe responsibly		✓			
Trauma and orthopaedics	Yes	The refractory prosthesis related infections with high resistance are a nightmare, and I think slowly increasing. Anything that might help is welcomed	✓		✓		
Vascular surgery	Yes	Widespread spread education and trials need to increase awareness.		✓			
Obstetrics and gynaecology	Yes	Really good BBC pod cast on phage therapy ’Crowd Science’				✓	
Obstetrics and gynaecology	Unsure	Don’t know anything about it or its use in my clinical practice		✓			
Medicine of the elderly	Yes	I attended the phage therapy grand rounds a few months ago and it seemed super interesting and promising! I love the idea particularly for the frailer patients with infected hips—they don’t do well on the months of antibiotics.	✓			✓	
Medicine of the elderly	Yes	Not sure that asking numbers is a great way of ascertaining needs—this is a new area within orthopaedics that I am not directly involved in, but would certainly consider in my patients whom had multi resistant infections in other areas that were not responding, however as a liaison service we would be directly referring to ID /Micro in these complex cases	✓		✓		✓
Obstetrics and gynaecology	Yes	This would need guidance and MDT discussion with our excellent Bacteriology colleagues					✓
Plastic surgery	Unsure	This is just something I read somewhere a while ago, but willing to explore due to the complex, infected wounds which we deal with, usually in conjunction with other specialties, e.g. ortho, neurosurgery, tissue viability	✓			✓	
Obstetrics and gynaecology	Unsure	Would need to learn more—what it is, safety, pros and cons, clinical indicators for use etc		✓			
Neonatology	Unsure	Much needed work	✓				
Renal medicine	Yes	Attended grand rounds on this a few months ago and I thought this may be useful in our chronic haemodialysis population with indwelling dialysis catheters, diabetics with vascular disease and in all our amputees with deep wound and bony infections	✓			✓	
Haematology	Yes	I have not kept up with the literature on phage therapy, so unsure whether the promise in clinical medicine that has been talked about for many years is finally coming to fruition.		✓		✓	
Dermatology	Yes	All sounds very interesting!					
Paediatrics	Unsure	Would be interested to know if there is any evidence base in neonates		✓			
Dermatology	Yes	My answers to 6 are rather subjective, and represent clinical issues facing one discipline, not an overview of the most important areas of development, seems to me that all listed organisms need new approaches to deal with resistance			✓		
Trauma and orthopaedics	Yes	This sounds like an excellent opportunity for orthopaedics. Particularly in cases with retained metalwork or resistant organisms. Would be great to have a Scotland wide service based in Tayside	✓		✓		
		**Totals**	13	11	6	6	4

ABR, antibiotic resistance.

## Discussion

In 2022 a Canadian group reported the results of a survey of 42 members of Association of Medical Microbiologists and Infectious Diseases Canada; this survey focussed on the experience of, and interest in, phage therapy in Canada [26]. More recently, an Australian group has published the results of a survey of 92 members of the Australian Society of Infectious Diseases; this survey examined awareness of phage therapy and perspectives on its development [27]. Here we report the findings of the first survey about phage therapy among clinicians in the UK. In contrast to previous surveys, this survey was conducted regionally, in two Scottish Health Boards, and sought opinions from clinicians of all medical specialties.

This survey demonstrated significant awareness of phage therapy among clinicians in Scotland, with 58.8% of respondents having previously heard of phage therapy. Elsewhere, high levels of awareness have also been observed among Australian infectious diseases and microbiology clinicians, although different question styles (yes/no vs. Likert scale) preclude direct comparison [27]. The level of awareness among Scottish clinicians is perhaps surprising, given the respondents were from the full breadth of medical specialties. However, there is significant interest in phage therapy in Scotland, especially with the recent Health Improvement Scotland recommendation in favour of phage therapy for difficult-to-treat infections [24]. Moreover, the survey was conducted in two Scottish Health Boards which have experience of using phage therapy. These factors have confounding potential and could contribute to an overestimation of wider clinical interest in phage therapy.

Phage therapy has the potential to transform the treatment of bacterial infections. Presently, in the UK phage therapy may be considered for use as an unlicensed medicine in cases of special clinical need [25]. This represents small numbers of unique and often complex cases, potentially drawn from any medical specialty. Consequently, obtaining prevalence data about the potential number of patients that could benefit from the expanded use of unlicensed phage therapy is challenging, if not impossible. Nevertheless, it is important for policymakers to appreciate the potential magnitude of impact that expanded unlicensed phage use could have. Notwithstanding prevalence data, this survey sought estimates of potential patient numbers from clinicians. This revealed substantial demand, with 300 potential phage patients seen among 77 respondents in the past 12 months. Encouragingly, most respondents reported already being willing to consider using phage therapy (71.1%). This may reflect confidence imparted by the Health Improvement Scotland recommendation [24]. Although this survey did not directly gather data about the rationale of clinicians who were not willing to use phage therapy, seven respondents who were unsure about using phage therapy stated or implied in the additional comments section that they would like more information about phage therapy. This is an appreciable concern among clinicians. While guidance, such as that from Health Improvement Scotland will support clinicians, it is important for clinicians to make their own assessments and draw on reliable sources of information and experience to inform their practice.

The breadth of demand across medical specialties illustrates the potential scope of impact. High demand across 11 Scottish Health Boards has previously been observed, although in that case clinicians were asked to estimate demand from their speciality in their hospital, rather than the lower per clinician estimations obtained by this survey [24]. The previous estimation revealed significant demand from diabetic foot and orthopaedic infections. In contrast, the results of this survey suggest significant demand from diabetes, respiratory medicine and paediatrics. As illustrated by the notable demand from paediatrics observed in this survey, the specialties of the respondents will confound interpretation of the specialties in which phage therapy may have the greatest impact. However, surveys can yield surprising areas of potential demand, such as paediatrics, neonatology and obstetrics and gynaecology. Per clinician estimations were chosen for this survey because we considered estimations may be more precise if clinicians were asked to consider their own practice, rather than that of their department. Moreover, per clinician estimations also provide policymakers with a readily digestible perspective of the scale of potential impact. For example, the results of the estimations in this survey are from just 77 clinicians, with extrapolation across the NHS yielding significant patient numbers. Therefore, phage therapy, even as an unlicensed medicine, has the potential to deliver substantial savings to the NHS, not just because of high savings on individual patient care but because of the number of potential patients that could benefit. Health Improvement Scotland have previously found the economic case for phage therapy in the context of diabetic foot infection to be strong [24]. Elsewhere, it has been estimated that use of licensed phage therapy could save the UK’s NHS £120 million per year on diabetic foot infection and £60 million per year on hip and knee infections [28]. Health economic analyses in an Australian context have been similarly favourable [29].

Previous surveys have identified *Pseudomonas*, mycobacteria and *E*. *coli* as priorities for phage therapy [26, 27]. However, such surveys were undertaken among cohorts of infectious disease specialists. This survey’s broader clinical cohort revealed cross-specialty priorities to be staphylococci, *Pseudomonas* and *E*. *coli*. Although under-powered due to low sample size, the per-specialty analysis of organism priority for phage therapy development revealed perhaps unsurprising trends, such as *Burkholderia* being a concern for respiratory medicine and streptococci for dermatology. Taken together, the results of this, and previous surveys, suggest that the ESKAPE pathogens and *Mycobacteria* should be prioritised for the development of phage therapy. Arguably, the broad applicability of phage therapy means that where off-the-shelf phage cocktails are developed a per-organism rather than per-indication approach could be taken. However, while clinically appropriate, this approach may not align well with the traditional ‘per indication’ pharmaceutical model. The ESKAPE pathogens are strongly associated with antibiotic resistance and concerning for policymakers [1]. However, the development of phage products against these pathogens will enable the treatment of antibiotic resistant infections and reduce reliance upon conventional antibiotics.

Antibiotic resistance is associated with increased financial costs to healthcare providers and has been found to be a concern among patients in Scotland [30, 31]. This survey found that antibiotic resistance is also a concern among Scottish clinicians, although the magnitude of impact on clinical practice naturally varies by specialty. Together 83 clinicians estimated seeing 711 patients in the last year whose infections were refractory to antibiotics, delaying or preventing resolution of infection. This number was higher than the estimated 300 potential phage patients, likely because this included patients in whom resolution was delayed but achieved with current therapeutics or patients for whom other approaches (e.g. surgical) were required.

The findings of this survey are limited by its small sample size, especially when divided by specialties. While this does not prevent an appropriately caveated descriptive assessment of these data, which is itself valuable, the small sample size precludes a more rigorous statistical analysis. Moreover, while on the one hand the estimations obtained in this survey are just estimations and should be approached with caution, they are also the opinions of experienced clinicians. While previous surveys have focussed on infectious diseases and microbiology clinicians, we took a broader cross-specialty approach. However, with a low sample size, this means that the conclusions of this survey will be influenced by the specialties of the respondents. For example, the results of this survey could be biased towards the experiences of paediatricians, who represented the largest proportion of respondents. Similarly, important experiences from specialties not represented are absent. As described above, there is growing interest in phage therapy in Scotland and it is possible this may have contributed a degree bias. That respondents were encouraged to forward the survey onto colleagues to increase the response rate is a further possible source of bias that has the potential to have reinforced any underlying sample bias.

## Conclusion

We report the findings of the first survey about phage therapy among clinicians in the UK. These results from clinicians in two Scottish Health Boards show that antibiotic resistance remains a substantial and concerning challenge. There is a substantial breadth and scale of demand for phage therapy among clinicians. Current access to unlicensed phage therapy is limited and widening access could deliver substantial clinical and financial returns for health authorities.

## Supporting information

S1 File Survey. (PDF)

S2 File Survey responses. (XLSX)

## Abbreviations

GG&C: Greater Glasgow and Clyde
NHS: national health service
