# Supplementary material for: Phage therapy: Awareness and demand among clinicians in the United Kingdom
Source: PLoS One. 2023 Nov 13;18(11):e0294190. doi: 10.1371/journal.pone.0294190 (PMC10642789; doi:10.1371/journal.pone.0294190)
Supplement: S1 File — (PDF) [file pone.0294190.s001.pdf]

Phage therapy: awareness and demand in NHS GGC

About phage therapy

Bacteriophages (phages) are viruses which specifically infect bacterial cells and are unable to infect human cells.

Phage therapy is the use of phages to treat bacterial infection.

Used widely in the early 20th Century, the use of phages declined with the mass production of antibiotics.

A lab test is required to investigate if a phage can kill a bacterial isolate; known as phage sensitivity testing.

The antimicrobial resistance crisis is driving renewed interest in phage therapy.

Health Improvement Scotland has recommended that phage therapy may be used for difficult-to-treat infections.

About this survey

This survey:

- is for doctors of any grade in NHS Greater Glasgow and Clyde
- explores awareness of and demand for phage therapy
- is being undertaken as part of biomedical scientist registration portfolio
- will take approximately 4 minutes to complete
- Your feedback is anonymous and will help us scope demand for phage therapy.
- We may share data and feedback within NHS GG&C and in an academic publication but we will not include any information which could identify you.

Please tell us about you

1. **Grade:** *

Enter your answer

2. **Specialty:** *

Enter your answer

3. **Within your clinical practice, how much of a concern is antimicrobial resistance?**
(1 = least concern, 5 = most concern) *

1	2	3	4	5

4. **Estimate the number of patients you have treated in the last year whose infections have been refractory to antibiotics, delaying or preventing resolution of infection?** *

Enter your answer

5. **Prior to this survey, had you heard of phage (or bacteriophage) therapy?** *

- ◯ Yes
- ◯ No
- ◯ Unsure

6. **Rank the organisms below in order of highest to lowest priority for the development of phage therapy.** *

- *Acinetobacter baumanii*
- *Burkholderia* species
- *E. coli*
- *Enterococcus faecium* and *Enterococcus faecalis* (including VRE)
- *Klebsiella* species
- *Mycobacterium* species
- *Pseudomonas* species
- *Staphylococcus* species (including MRSA)
- *Streptococcus* species

7. **Are there any other organisms that you would like phage therapy to be developed for?**

Enter your answer

8. Phage therapy is not currently licensed in the UK, but phages may be prescribed as an unlicensed medicine in cases where licensed medicines (e.g. antibiotics) cannot meet the patient's clinical needs.

This includes cases with:

- antibiotic resistance
- antibiotic sensitivity but clinical recalcitrance
- high risk of death or significant complications if surgical intervention is used to manage their infection
- patient specific factors that preclude the use of conventional antibiotics.

Estimate of the number of patients you have treated in the last year that you think may have been eligible for phage therapy, regardless of bacterial species.

i.e. patients for whom antibiotics were not meeting their clinical needs. *

Enter your answer

9. **Would you consider phage therapy for a patient if antibiotics were not meeting their clinical needs?** *

- ◯ Yes
- ◯ No
- ◯ Unsure

10. **Additional comments**

Enter your answer
